# Downregulation of lncRNA‐HEIH curbs esophageal squamous cell carcinoma progression by modulating miR‐4458/PBX3

**DOI:** 10.1111/1759-7714.13489

**Published:** 2020-05-25

**Authors:** Dawei Wang, Dong You, Yinghua Pan, Peiji Liu

**Affiliations:** ^1^ Department of Radiotherapy Yantai Yuhuangding Hospital Affiliated to Qingdao University Yantai China; ^2^ Department of Radiology Yantai Yuhuangding Hospital Affiliated to Qingdao University Yantai China

**Keywords:** Esophageal squamous cell carcinoma, HEIH, miR‐4458, PBX3

## Abstract

**Background:**

Long non‐coding RNAs (lncRNAs) have been found to play a specific part in the development of esophageal squamous cell carcinoma (ESCC), except for lncRNA HEIH. Here, we aimed to discover the molecular mechanisms of HEIH in ESCC.

**Methods:**

We detected the expression level of HEIH and miR‐4458 in ESCC tissues and cells using qRT‐PCR assay. A dual luciferase reporter assay was used to check the relationship between HEIH, miR‐4458 or PBX3. Counting Clock Kit‐8 (CCK‐8) assay and transwell assay were used to detect ESCC cell proliferation and invasion capability. Western blot analysis was used to measure the protein expression level of PBX3.

**Results:**

HEIH was confirmed to be upregulated in both ESCC tissues and cell lines. Inversely, there was a downregulation of miR‐4458 in ESCC tissues and cell lines. Functionally, we noticed that depletion of HEIH restrained ESCC cell viability, and invasion capability. Moreover, PBX silencing was found to restrain ESCC cell progression, while miR‐4458 or HEIH vector both could alleviate its suppressive effect.

**Conclusions:**

The present study clarified that HEIH regulated ESCC progression by suppressing miR‐4458 and upregulating PBX3. Our findings suggested that HEIH could be a possible therapeutic target for ESCC treatment.

## Introduction

Esophageal cancer (EC) is one of the commonest malignant tumors of the digestive system. According to statistics, the incidence rate and mortality rate of EC are eighth and sixth in the world, respectively, and with an obvious upward trend.^1^ Most esophageal cancers are esophageal squamous cell carcinoma (ESCC) which accounts for more than 90% of EC, and with high aggressiveness and complex biological behavior. ESCC is mainly caused by risk factors such as smoking, drinking or nutritional imbalance.^2^ Currently, ESCC is treated with surgery, chemotherapy or radiotherapy, which significantly improves the survival time of patients. However, there are no significant clinical symptoms in the early stage of ESCC. As a result, the five‐year survival rate of patients with advanced ESCC is less than 30%.^3^ Therefore, it is important to study the pathogenesis of ESCC and find early diagnosis markers and new treatment methods to improve the survival time of ESCC patients.

Long non‐coding RNAs (lncRNAs) are a class of RNA molecules longer than 200 nucleotides. Recent studies have found that multiple LncRNAs can participate in a variety of molecular and cellular processes, such as chromosome dosage compensation, imprinting, intracellular transport, cell cycle regulation, reprogramming, epigenetic regulation, stem cell differentiation, and regulate a variety of developmental processes of human diseases. In recent years, more and more lncRNAs have been found to be differentially expressed in tumors, and serve as a crucial part in the tumor genesis and development. In ESCC, CASC9, ECM, and OIP5‐AS1 are demonstrated to enhance ESCC cell metastasis and could be biomarkers for ESCC prognosis and treatment.^4^, ^5^, ^6^ Furthermore, Chang *et al*.^7^ verified that TUSC7 restrained cell proliferation and chemotherapy resistance and colony formation in ESCC. LncRNA HEIH was first reported to be upregulated in hepatocellular carcinoma.^8^ Moreover, HEIH was observed to facilitate cell proliferation, invasion and migration capability in melanoma.^9^ Guo *et al*.^10^ found that upregulation of HEIH enhanced chemoresistance of paclitaxel, and promoted cell viability by activating the MAPK pathway in endometrial cancer. As far as we know, there are no reports on the relationship between HEIH expression and the cell progress in ESCC.

LncRNAs serve as ceRNAs of microRNAs (miRNAs) to directly regulate target genes. At present, great progress has been made in the study of miRNAs, which are found participate in cell proliferation, migration, apoptosis, invasion, gene regulation and disease regulation. MiR‐375 has been identified as a new prognosis marker in ESCC.^11^ In addition, miR‐92a‐3p has been found to accelerate cell progression of ESCC cells by inhibiting PTEN.^12^ MiR‐4458 has been reported to be a tumor‐inhibiting factor in hepatocellular carcinoma.^13^ In breast cancer (BC), miR‐4458 was downregulated in BC tissues, and upregulation of miR‐4458 restrained cell proliferation, invasion and migration by regulating CPSF5.^14^ Nevertheless, the role of miR‐4458 in ESCC has still not been investigated.

In this article, we aimed to explore the expression level of HEIH and miR‐4458 in ESCC tissues and cells. Moreover, the role of HEIH/miR‐4458/PBX3 axis on ESCC cell progression in vitro was also detected.

## Methods

### Patients

A total of 48 ESCC tissues and the corresponding paracancer normal tissues (3–5 cm from the primary tumor) were collected from patients at the Yantai Yuhuangding Hospital Affiliated to Qingdao University. All ESCC patients in this study had not received any preoperative radiotherapy or chemotherapy. The clinical samples were stored in the refrigerator at −80°C for extraction of total RNA. All patients signed informed consents before the study commenced. The experiment was approved by the ethics committee of Yantai Yuhuangding Hospital Affiliated to Qingdao University.

### Cell lines and cell transfection

ESCC cell line Kyse150 and normal ESCC cell lines Het‐1A were obtained from Shanghai EK‐Bioscience Biotechnology Co., Ltd. (Shanghai, China). EC9706 and Yes‐2 were purchased from BeNa Culture Collection (Beijing, China). ESCC cells were seeded in RPMI1640 medium containing 10% FBS at 37°C with 5% CO_2_, and the medium was changed every 24 hours. When the cell confluence reached 80%, 0.25% trypsin was used for cell passage.

Si‐HEIH, miR‐4458 inhibitor, si‐PBX3, pcDNA3.1‐HEIH, miR‐4458 mimics and pcDNA3.1‐PBX3 were obtained from GenePharma compary. Lipofectamine 2000 was used to perform cell transfection.

### Real‐time quantitative PCR

Total RNA was isolated from ESCC tissues and ESCC cell lines according to TRIzol reagent instructions, and then reversely transcribed into cDNA using the special RT‐PCR Kit. Then, LightCycle 96 thermocycle was adopted to quantify the expression level of HEIH, miR‐4458 and PBX3. The expression of HEIH, miR‐4458 and PBX3 were analyzed by the 2^‐ΔΔCt^ method. HEIH and PBX3 were standardized to GAPDH, while β‐actin was normalized to miR‐4458. The primer sequences are listed in Table 1.

**Table 1:** Primer sequences in qRT‐PCR

Gene		Primers5′‐3′
HEIH	Forward	5′‐ATGCGAGAAGCCATGAGACC‐3′
	Reverse	5′‐GGAACAGCTTGTGTGACCGA‐3′
miR‐4458	Forward	5′‐AGAGGTAGGTGTGGAAGAA‐3′
	Reverse	5′‐GCGAGCACAGAATTAATACGAC‐3′
PBX3	Forward	5′‐GACATCGGCGACATCCTCC‐3′
	Reverse	5′‐TCACACAGGACGCTGAAGAG‐3′
GAPDH	Forward	5′‐CTCTGCTCCTCCTGTTCGAC‐3′
	Reverse	5′‐GACTCCGACCTTCACCTTCC‐3′
β‐actin	Forward	5′‐CCTGGCACCCAGCACAAT‐3′
	Reverse	5′‐GCTGATCCACATCTGCTGGAA‐3′

### Dual luciferase reporter assay

EC109 cells were grown at 37°C, 5% CO_2_ and 95% humidity. After 48 hours of incubation, the luciferase activity of HEIH and PBX3 were estimated by Dual‐Luciferase reporter assay kit.

### Cell Counting Kit‐8 (CCK‐8) assay

EC109 cells were seeded in a 96‐well plate at 37°C with 5% CO_2_. After culture for 24, 48, 72 and 96 hours, CCK‐8 reagents were added into each well. Finally, the relative optical density (OD) was detected with a value of 450 nm.

### Transwell assay

For cell invasion, cells (1 × 10^4^/well) were detected in the upper chamber with Matrigel. For cell migration, EC109 cells were seeded in DMEM without serum, and added to the upper chamber without Matrigel. Then, 500 μL DMEM (10% FBS) was added to the lower chamber. After incubation at 37°C for 24 hours, cells were stained with 0.1% crystal violet for 20 minutes. Finally, the number of migrated or invaded cells were counted under an optical microscope.

### Western blot assay

Protein samples were separated by 10% SDS‐PAGE, and then transferred to NC membrane. After incubating with 5% nonfat milk, the membranes were incubated with primary antibodies overnight at 4°C. The primary antibodies were as followed: PBX3 (polyclonal rabbit; cat. No. 12571‐1‐AP; 1:500) and β‐actin (Santa Cruz;1:500). Enhanced chemiluminescence (ECL) western blotting substrate (Thermo Fisher Scientific Inc., Waltham, MA, USA) was used to detect the protein signals. β‐actin was used as the control.

### Statistical analysis

All data were shown as mean ± standard deviation. GraphPad Prism 8 and SPSS 19.0 were performed to analyze the results. The statistical differences between two groups were assessed by Student's *t*‐test. The differences of multiple groups were analyzed by one‐way ANOVA. The correlation between HEIH, miR‐4458 and PBX3 were explored by Pearson's correlation analysis. *P* < 0.05 was considered as statistically significant.

## Results

### Expression of HEIH in ESCC and its relationship with clinicopathological characteristic of ESCC patients

**Figure 1:**
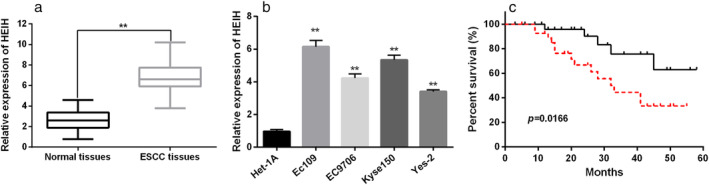
The expression of HEIH in esophageal squamous cell carcinoma (ESCC) and its relationship with clinicopathological characteristic of ESCC patients. (**a**) The expression of HEIH in ESCC tissues. (**b**) The expression of HEIH in ESCC cell lines (EC109, EC9706, Kyse150 and Yes‐2) and normal ESCC cells Het‐1A. (**c**) The survival time of ESCC patients in high HEIH expression group and low HEIH expression group (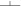 Low HEIH expression and 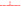 High HEIH expression). **P < 0.01.

First, we explored the expression of HEIH in ESCC tissues by qRT‐PCR analysis. The results revealed that there was an ascended expression of HEIH in ESCC tissues compared to that in normal tissues (Fig 1a). Afterwards, we detected the expression of HEIH in four ESCC cell lines: EC109, EC9706, Kyse150 and Yes‐2. The results showed that HEIH was highly expressed in ESCC cell lines (EC109, EC9706, Kyse150 and Yes‐2) than in the normal ESCC cell line Het‐1A (Fig 1b). Based on the average value of HEIH expression, we divided 48 ESCC patients into the high HEIH expression group and low HEIH expression group. We found that ESCC patients in the high HEIH expression group had lower survival time than those in the low HEIH expression group (Fig 1c). Furthermore, high HEIH expression was closely related to tumor grading and TNM stage (Table 2). All results indicated that HEIH participated in the progression of ESCC.

**Table 2:** Correlation between the expression level of HEIH and clinical characteristics of esophageal squamous cell carcinoma (ESCC) patients (*n* = 48)

		HEIH expression		
Clinical characteristics	Number of cases (*n* = 48)	Low (*n* = 20)	High (*n* = 28)	*P*‐value
Age (years)				0.203
≤60	22	7	15	
>60	26	13	13	
Gender				0.461
Male	27	10	17	
Female	21	10	11	
Tumor size				0.406
≤4 cm	23	11	12	
>4 cm	25	9	16	
Tumor location				0.113
Upper/middle	28	9	19	
Lower	20	11	9	
Tumor grading				0.041*
G1	14	9	5	
G2/3	34	11	23	
TNM stage				0.003**
<III stage	17	12	5	
≥III stage	31	8	23	

* *P* < 0.05, the difference is significant.

** *P* < 0.01, the difference is highly significant.

### Knockdown of HEIH suppressed cell proliferation and invasion in ESCC

**Figure 2:**
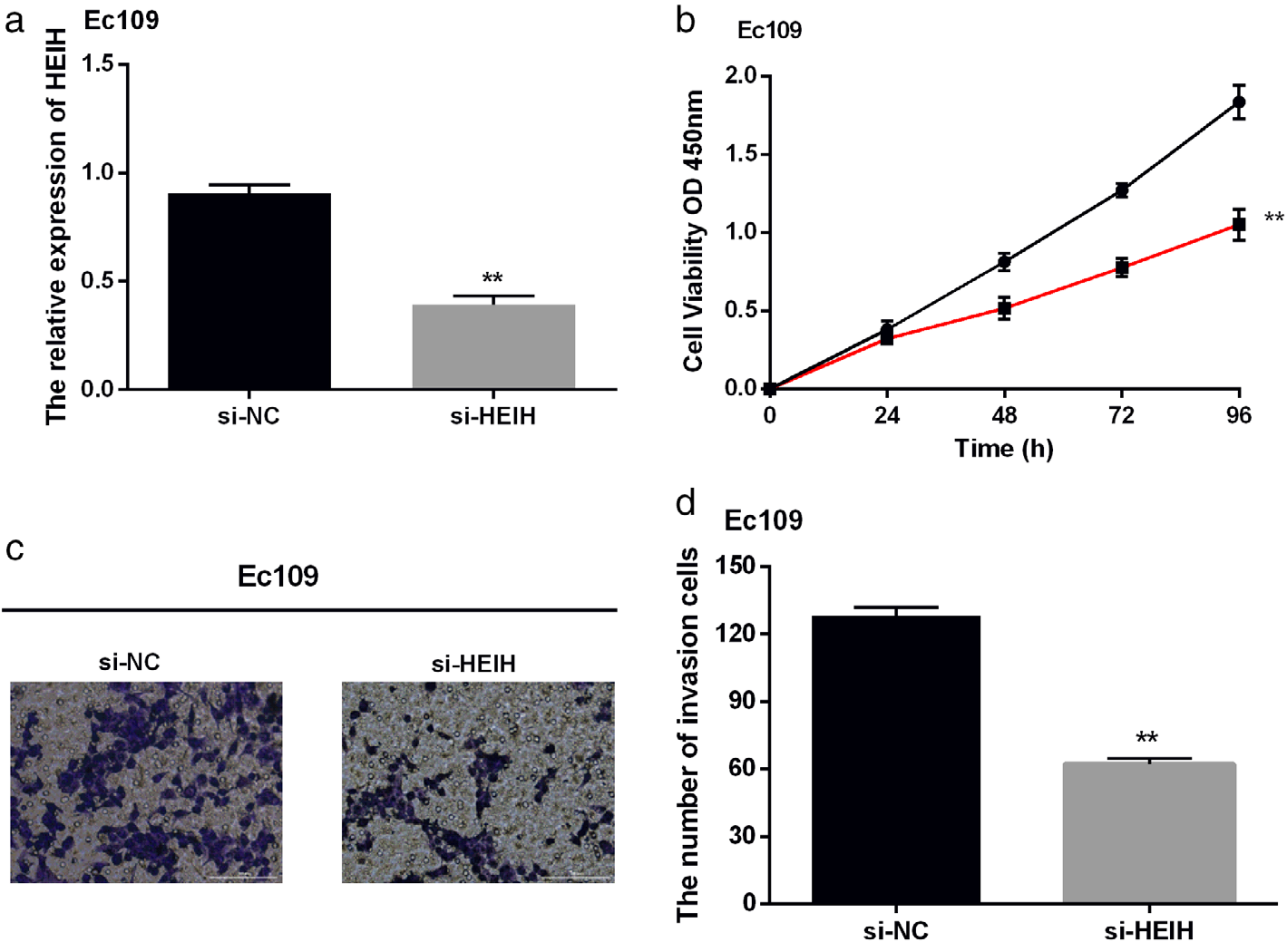
Knockdown of HEIH suppressed cell proliferation and invasion in esophageal squamous cell carcinoma (ESCC). (**a**) The expression of HEIH was decreased by si‐HEIH. (**b**) Cell proliferation of EC109 cells was inhibited by si‐HEIH (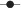 si‐NC and 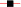 si‐HEIH). (**c**) Cell invasion of EC109 cells was suppressed by si‐HEIH (magnification: 20×). **P < 0.01.

To investigate the specific function of HEIH in the progression of ESCC, CCK‐8 and transwell assays were carried out. According to the results, EC109 cells can represent ESCC cells for follow‐up functional experiments. The knockdown of HEIH was transfected into EC109 cells, and we found that the expression of HEIH was obviously decreased (Fig 2a). Functionally, cell proliferation was found to be suppressed by silencing HEIH (Fig 2b). Similarly, knockdown of HEIH decreased the invasion of EC109 cells (Fig 2c). Our results illustrated that silencing of HEIH suppressed cell proliferation and invasion of EC109 cells.

### HEIH served as a molecular sponge of miR‐4458 in ESCC

**Figure 3:**
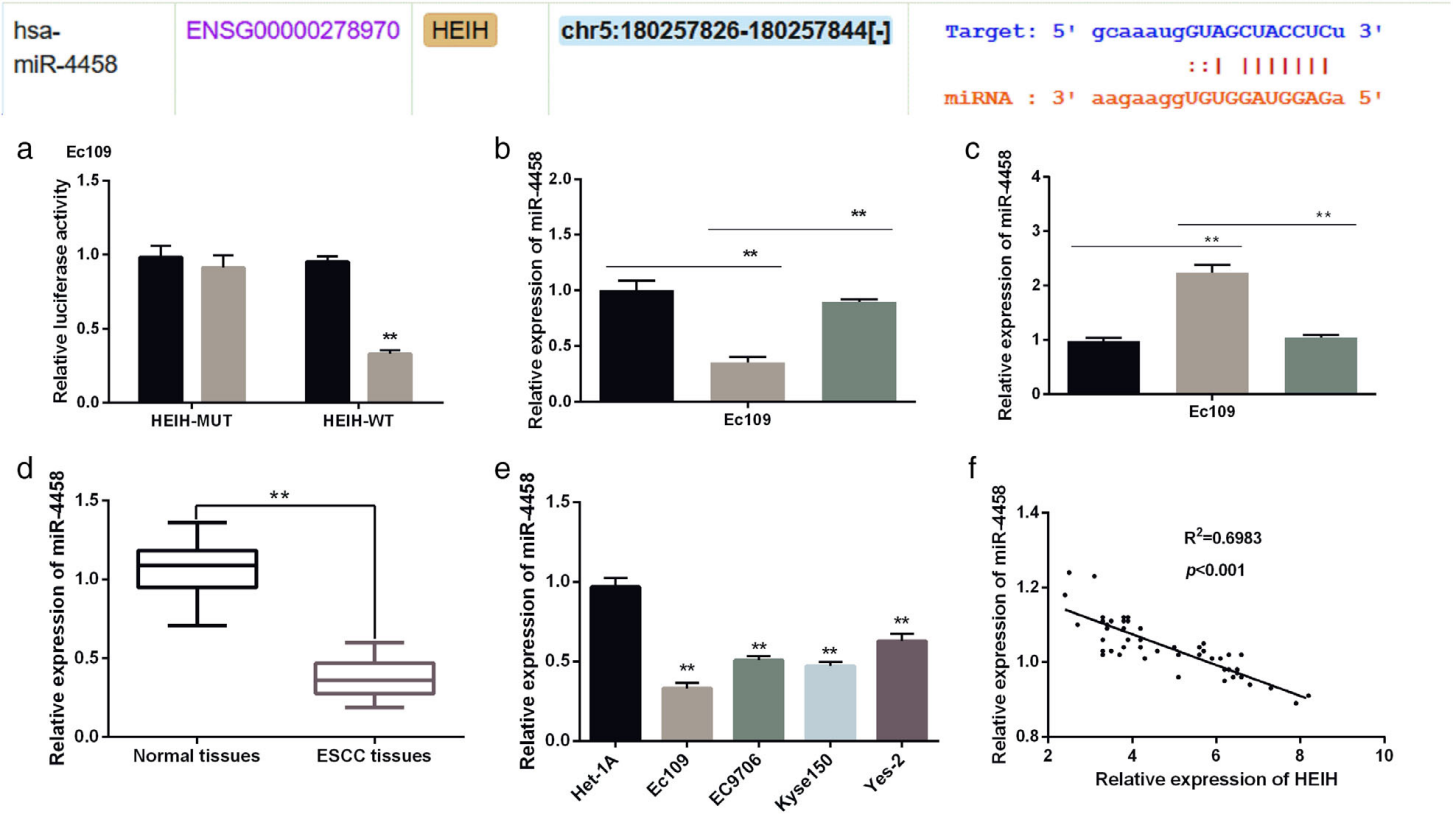
HEIH served as a molecular sponge of miR‐4458 in esophageal squamous cell carcinoma (ESCC). (**a**) There were binding sites between HEIH and miR‐4458. (**b**) The luciferase activity of HEIH –WT and HEIH‐MUT (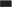 miR‐NC and 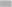 miR‐4458). (**c**) The expression of miR‐4458 in EC109 cells (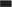 NC, 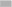 HEIH vector, and 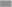 HEIHvector+miR‐4458 mimics). (**d**) The expression of miR‐4458 in ESCC tissues (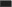 si‐NC, 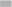 si‐HEIH, and 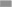 si‐HEIH+miR‐4458 inhibitor). (**e**) The expression of miR‐4458 in ESCC cell lines. (**f**) There was a negative correlation between HEIH and miR‐4458. **P < 0.01.

HEIH has been reported to act as ceRNAs of miRNAs in several human cancers. In our study, StarBase was performed to search miRNAs which had binding sites with HEIH in ESCC. The results displayed that there were specific binding sites between HEIH and miR‐4458 (Fig 3a). To verify this hypothesis, miR‐4458 mimics were transfected into EC109 cells. The results showed that the luciferase activity of HEIH‐WT was decreased by miR‐4458 mimics, while there was no change in HEIH‐MUT (Fig 3b). HEIH vector was transfected into EC109 cells with miR‐4458 mimics. As shown in Fig 3c, upregulation of HEIH decreased the expression level of miR‐4458, while it was reversed by miR‐4458 mimics. Moreover, the expression of miR‐4458 was increased by HEIH silencing, while recovered to homologous controls by miR‐4458 inhibitor (Fig 3d). Next, we explored the expression of miR‐4458 in ESCC tissues and cell lines. We found that miR‐4458 was notably downregulated in ESCC tissues (Fig 3e). Moreover, we found that the expression level of miR‐4458 was low in ESCC cell lines compared with Het‐1A cells (Fig 3f). Pearson's correlation analysis was adopted to analyze the relationship between HEIH and miR‐4458. Our results showed that HEIH was negatively correlated with miR‐4458 (Fig 3g). Combined with the above results, HEIH acted as a molecular sponge of miR‐4458 in ESCC.

### PBX3 was a target gene of miR‐4458 in ESCC

**Figure 4:**
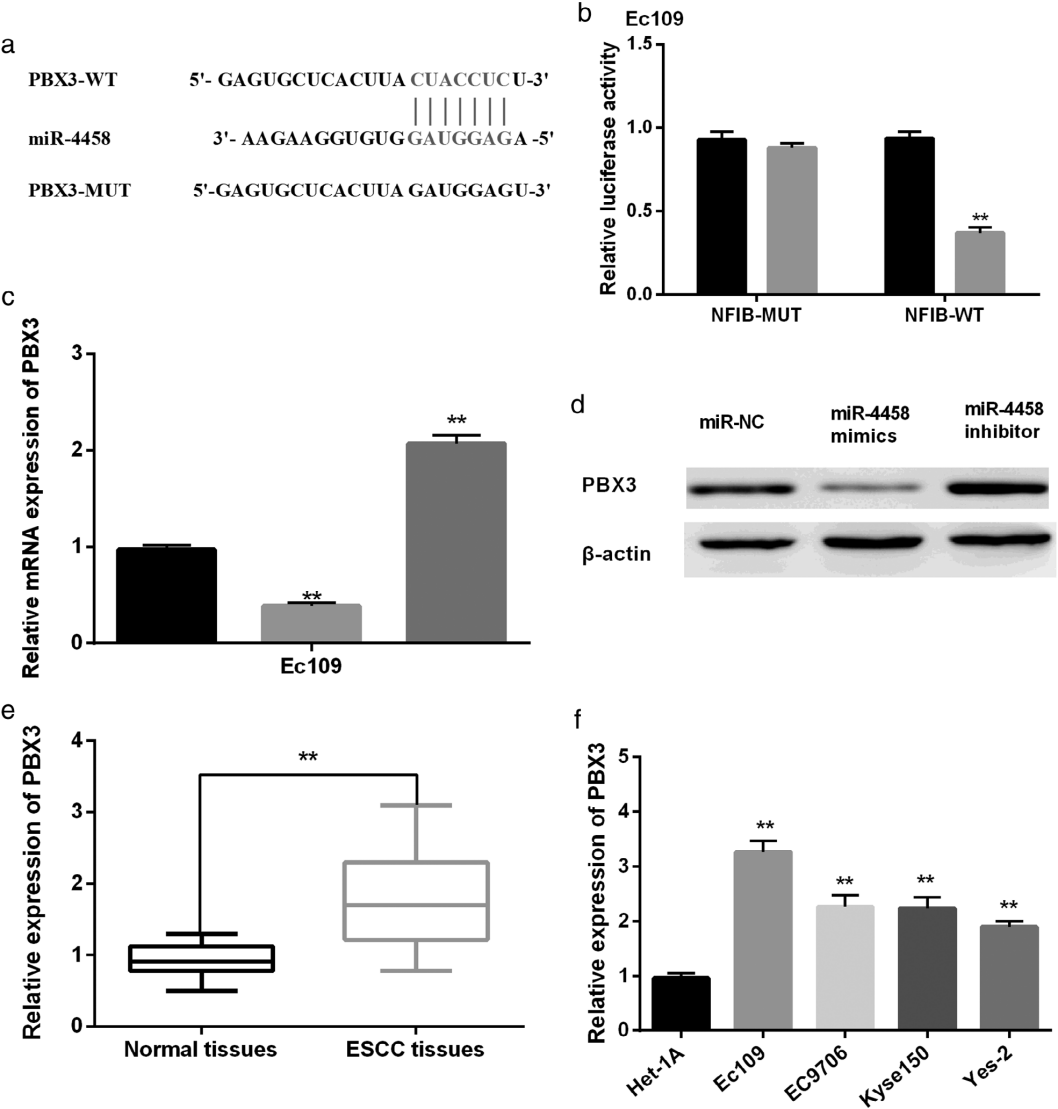
PBX3 was a target gene of miR‐4458 in esophageal squamous cell carcinoma (ESCC). (**a**) MiR‐4458 had binding sites with PBX3. (**b**) The luciferase activity of PBX3‐WT and PBX3‐MUT (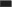 miR‐NC and 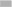 miR‐4458 mimics). (**c**) The mRNA expression of PBX3 (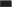 NC, 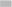 miR‐4458 mimics, and 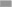 HEIH vector). (**d**) The protein expression of PBX3. **P < 0.01.

Next, TargetScan software was applied to search the target genes for miR‐4458. We noticed that there were binding sites between miR‐4458 and PBX3 (Fig 4a). Moreover, as shown in Fig 4b, the luciferase activity of PBX3‐WT was reduced by miR‐4458 mimics, while there was almost no change in PBX3‐MUT. To explore the relationship between miR‐4458 and PBX3, EC109 cells were transfected with miR‐4458 mimics or miR‐4458 inhibitor, respectively. We found that the mRNA expression of PBX3 was reduced by miR‐4458 mimics, but enhanced by miR‐4458 inhibitor (Fig 4c). Moreover, miR‐4458 was found to have the same effect on protein expression of HEIH (Fig 4d). We noticed that PBX3 was significantly upregulated in ESCC tissues and cell lines (Fig 4e,f). All data indicated that PBX3 was a target gene of miR‐4458 in ESCC.

### HEIH modulated ESCC cell progression via regulating miR‐4458/PBX3 axis

**Figure 5:**
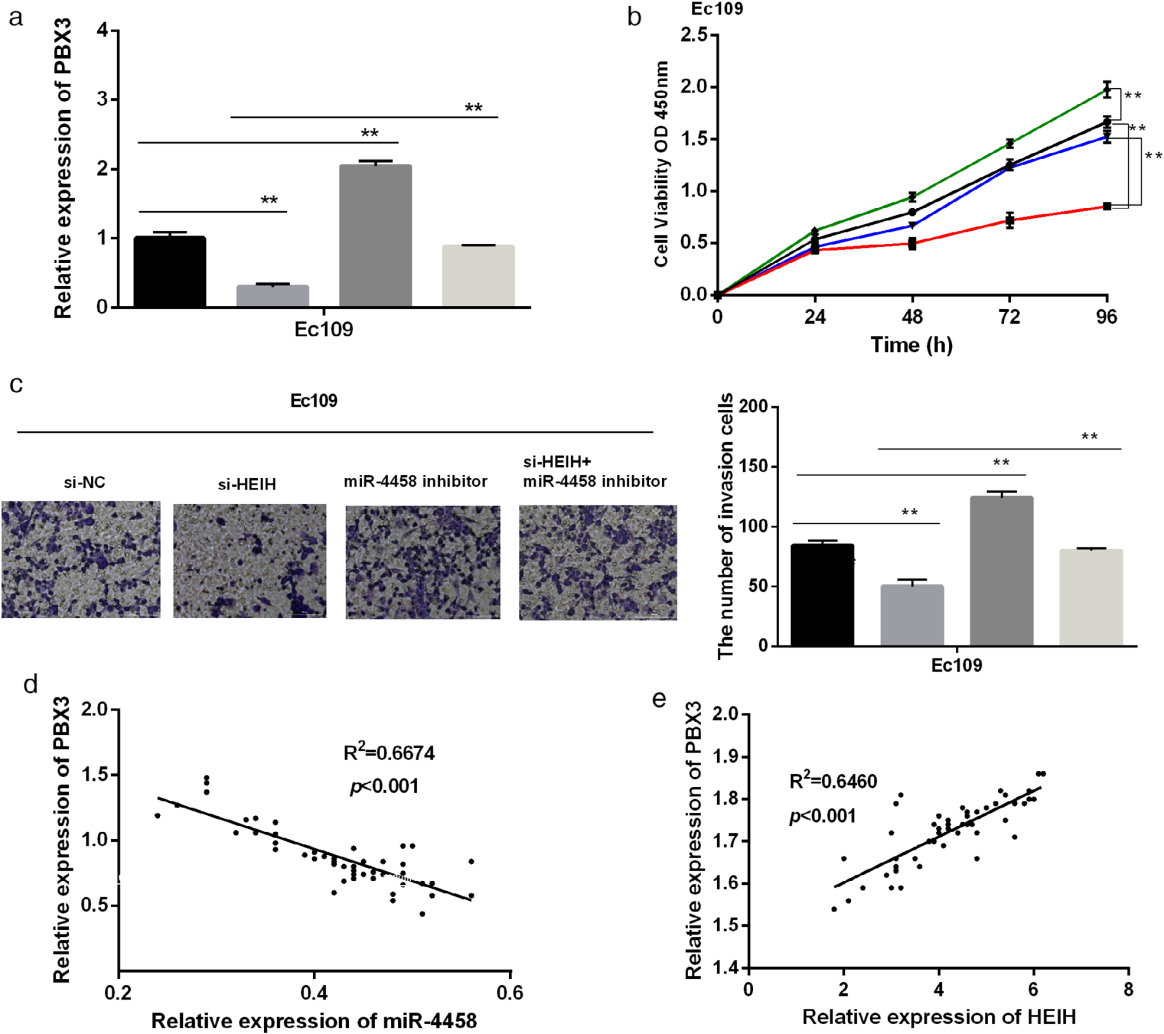
HEIH modulated esophageal squamous cell carcinoma (ESCC) cell progression via regulating miR‐4458/PBX3. (**a**) The expression of PBX3 (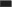 si‐NC, 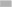 si‐HEIH, 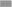 miR‐4458 inhibitor, and 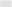 si‐HEIH+miR‐4458 inhibitor). (**b**) Cell proliferation was inhibited by si‐PBX3, while miR‐4458 or HEIH vector destroyed the inhibition (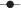 si‐NC, 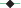 si‐HEIH, 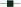 miR‐4458 inhibitor, and 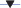 si‐HEIH+miR‐4458 inhibitor). (**c**) Cell invasion was suppressed by PBX3 silencing, while increased by miR‐4458 or HEIH vector (magnification: 20×) (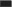 si‐NC, 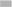 si‐HEIH, 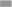 miR‐4458 inhibitor, and 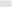 si‐HEIH+miR‐4458 inhibitor). (**d**) PBX3 was negatively correlated with miR‐4458. (**e**) PBX3 was positively correlated with HEIH. **P < 0.01.

To examine whether HEIH modulated ESCC cell progression through miR‐4458/PBX3, HEIH knockdown or miR‐4458 inhibitor was transfected into EC109 cells. RT‐qPCR results revealed that the expression of PBX3 was reduced by si‐HEIH and enhanced by miR‐4458 inhibitor, while the cotransfection of si‐HEIH with miR‐4458 inhibitor neutralized the adverse effect induced by si‐HEIH (Fig 5a). CCK‐8 assay showed that miR‐4458 inhibitor promoted cell proliferation of EC109 cells, while si‐HEIH cotransfected with miR‐4458 inhibitor recovered the inhibition effect of si‐HEIH on cell proliferation (Fig 5b). Similarly, depletion of miR‐4458 had the same reversion effect on cell invasion affected by si‐HEIH (Fig 5c). In addition, we discovered that there was a negative correlation between PBX3 and miR‐4458, while PBX3 was positively correlated with HEIH (Fig 5d,e). Hence, we concluded that HEIH modulated ESCC cell progression by regulating miR‐4458‐mediated PBX expression.

## Discussion

At present, the study of the role on the abnormal expression of LncRNAs in the tumor genesis, metastasis and diagnosis is a hot topic. EGFR‐AS1,^15^ MNX1‐AS1^16^ and FER1L4^17^ have been found to have influences on tumorigenesis and development in ESCC. Scientists have previously found that lncRNA HEIH plays a carcinogenic role in colorectal cancer^18^ and non‐small cell lung cancer.^19^ In this study, we noticed that HEIH was markedly elevated in ESCC tissues and cells, which was consistent with the expression trend in previous studies. Next, CCK‐8 assay and transwell assay were performed to detect the molecular function of HEIH in the progression of ESCC cells. Functional experiments revealed that silencing of HEIH restrained cell proliferation and invasion of EC109 cells. In breast cancer, silencing of HEIH suppressed cell proliferation and metastasis but enhanced cell apoptosis, which was similar to our results.^20^ In addition, we found that HEIH was closely correlated with tumor grading and TNM stage. Our findings indicate that HEIH has a great potential to be a treatment target for ESCC.

LncRNAs have been proved to competitively bind with miRNAs, and to participate in tumor regulation by inhibiting miRNAs expression. Masayuki *et al*. screened out 15 downregulated miRNAs of the 365 miRNAs in ESCC tissues.^21^ In cholangiocarcinoma, Wan *et al*. found HEIH exhibited a positive role in regulating cell proliferation, invasion and migration by inhibiting miR‐98‐5p and upregulating HECTD4.^22^ In our study, we speculated that HEIH interacted with miR‐4458. In triple‐negative breast cancer, HEIH was also found to regulate cell viability by competitively binding with miR‐4458l.^23^ The results revealed that HEIH vector reduced the expression level of miR‐4458. Moreover, there was a negative relationship between HEIH and miR‐4458. Therefore, HEIH was confirmed to act as a molecular sponge of miR‐4458 in ESCC.

Preleukemia transcription factor 3 (PBX3) is part of the PBX family, and has been found to be a tumor‐promoting gene in several human tumors, such as cervical cancer^24^ and papillary thyroid carcinoma.^25^ We confirmed that PBX3 was a target gene of miR‐4458. In addition, we found that miR‐4458 mimics reduced the mRNA and protein expression of PBX3, while miR‐4458 inhibitor inhibited PBX3 expression. Parallel with our findings, PBX3 was also found to be regulated by miR‐4458 in tumorigenesis of melanoma.^26^ Furthermore, silencing of PBX3 inhibited cell proliferation and invasion of EC109 cells, while miR‐4458 mimics or HEIH vector both migrated the inhibitory effects stimulated by si‐PBX3. Consistent with previous results, PBX was revealed to enhance cell proliferation, migration and invasion in gastric cancer.^27^ Moreover, our findings indicated that HEIH regulated ESCC cell progression by modulating the miR‐4458/PBX3 axis.

In conclusion, HEIH was first found to be notably elevated in ESCC tissues and cells. We confirmed that HEIH silencing inhibited cell proliferation and invasion of ESCC cells. Moreover, our results confirmed that HEIH regulated ESCC cell progression by sponging miR‐4458 and upregulating PBX3. Therefore, the study of the biological function of HEIH can provide new ideas for the diagnosis and treatment of ESCC in the future.

Disclosure

The authors confirm that there are no conflicts of interest.
